# Points from Hospital Reports

**Published:** 1924-07

**Authors:** 


### POINTS FROM HOSPITAL REPORTS.

### Birmingham General Hospital.

The work of 1923 was very heavy, the number of in-patients being 6,153, and the daily average in the wards 314, compared with 5,773 and 297 in 1922. Out-patients numbered 47,696 against 42,041. The total expenditure was greater by £6,524 than in 1922, but the total income was more than sufficient to meet it. The annual subscription list is of very impressive dimensions—£16,430—and the following analysis of it quoted from the Report is even more so:—Private subscriptions, £2,304; Business subscriptions, £6,281; Workpeople's subscriptions, £7,845 That business houses, master and man, and the man the greater part, should contribute regularly to the Hospital treasury more than £14,000 a year—one-fifth of the annual expenditure—may well inspire the administration with confidence. The need for expansion is making itself felt, and if negotiations which are in progress for the acquisition of a site near to the Hospital are successful, the Board will be making definite recommendations to the Governors on the subject. It would enhance the interest of the statistical table on pages 102 to 104 if a column for the number of beds available were added. The growth in the number of patients from year to year is interesting, but, side by side with it, the periodical increase in the number of beds to meet it is equally, or even more interesting, to those who look ahead.

### Harrogate Infirmary.

An increase of work and of income and a decrease of expenditure is a statement which conveys in a few words a general idea of the progress of this Infirmary during 1923. Analytical tabular statements of income and expenditure supply the very useful and rather uncommon information that the average income per bed amounted to £154, and the expenditure to £148, which is much more useful knowledge than the mere statement that the income exceeded the expenditure by so much. Indeed, the statistical tables generally on pages 14 to 20 are highly to be commended. We do not, however, find the number of beds available stated: we compute it at 55. One of the most encouraging items in the whole financial survey is the increase in the amount of annual subscriptions, and another, perhaps even more satisfactory, is the continued growth of patients' contributions. While we warmly endorse the Committee's view that the best way to attract public interest and support is to do what they have done, go out among supporters—in clubs, halls, and meeting places of one kind and another—and lay before their hearers clear statements of the work and consequent financial needs of the Infirmary, we think their statement that appeals and circulars are valueless is too sweeping. All who should be approached cannot be reached by harangues, however eloquent, at meetings, which to some people are even more distasteful than circulars. People who do not attend meetings—and they are a vast majority—can only be reached by the post, but the appeals must be concise, earnest and telling, and not the slovenly, unattractive generalities and fustian that they often are. The table of contents might be improved by alphabetical arrangement, expansion and revision, the valuable statistics of which we have just spoken, for example, are not included in it.

### Royal Portsmouth Hospital.

In consequence of the establishment of a hospital at Gosport, this institution, formerly known as the Royal Portsmouth, Portsea and Gosport Hospital, is henceforth to be called by the above name. Owing largely to the special efforts of the Southsea Carnival and Mayor's Charities Committees during the past two years, which have resulted in the receipt by the hospital of no less than £10,000, it is now out of debt. The "ticket system," which often dies hard because of that trait of human nature which, even in the domain of charity, cannot always bring itself to give without the prospect of material return, was abolished here during the year. In this connection the Committee say:—"It is 'up to' the public to subscribe just as freely—tickets or no tickets—and to recognise that to have no tangible return in the shape of tickets means the admission of patients with less hardship and inconvenience than under the old system." A new mortuary and mortuary chapel have been provided during the year by the munificence of Sir Woolmer White, Bart. The hospital contains 166 beds, and although the average number occupied during 1923 was 3 higher than in 1922, and 169 more in-patients were treated, there was a slight reduction (£328) in expenditure. As usual, the obdurate department was "Surgery and Dispensary," in which there was an increase of £632. The ordinary income stands in need of development, for although the total income exceeded the total expenditure by £692, the ordinary income fell short of the cost of maintenance and administration by more than £3,000.

### Minehead and West Somerset Hospital.

It is very pleasant to find this little hospital of forty-four beds so full of vitality. The Report is a most interesting document, which affords ample internal evidence of the possession, on the part of the Committee and medical staff, of qualities which augur well for the future progress of the institution. In particular we are edified by the evidently cordial relations which subsist between the medical staff and the lay committee. The judgment and business acumen shown by the adoption of the policy of enlisting special interest in particular developments deserve a word of recognition. Is a new wing necessary? The case is stated, and Mr. R. D. Magor comes forward, provides practically the whole sum necessary, and "The Magor Wing" is the result. Is a complete X-ray installation needed? The same course is followed. Result: £640 in hand, and this year will see the want supplied. Now an electric lift is required. The case is put, and—it is scarcely appropriate to say, "Wait and see," for is not the thing a foregone conclusion? It is fitting, but not the less gratifying on that account, to see the names Luttrell and Acland—names to conjure with in this delightful *Lorna Doone* country—prominently associated with the hospital, which is styled "The Luttrell Memorial." We wonder whether the Mr. J. Ridd of Oare, who conducted a whist drive which put £16 into the hospital's coffers, is a descendant of Blackmore's redoubtable and lovable hero? But surely there can be no doubt about it!

### Mildmay Memorial Hospital.

This hospital of thirty beds had a very busy year in 1923, the number of in-patients, 273, exceeding by practically a third that of the year 1922. The expenditure, as might be expected, was somewhat heavier. The statistical tables show that while the average cost per in-patient per week was inappreciably higher—£3 11s. 11d. against £3 10s. 10d.—that of the total cost per head was, by reason of the larger number treated and the shorter period of stay, of course much less—£14 8s. 8d., compared with £18 2s. 3d. This is quite a useful illustration of the fact that, as overhead charges in any given hospital are practically stationary, it is good economically and statistically—in short, practically—that its beds should be kept fully occupied throughout the year. It also shows how fallacious it is to talk of reducing expenditure by closing beds. Nothing approaching proportionate reduction can be brought about by such means. There was a small excess, at the Mildmay Memorial, of expenditure over income. The year was not marked by any prominent event.

### Providence Hospital, Washington, D.C.

This, the oldest and largest Hospital in the capital of the United States, was primarily a war hospital, and was founded by the Sisters of Charity of St. Vincent de Paul of Emmitsburg, Maryland, in 1861, in the early days of the Civil War. It has been conducted by the Sisters ever since. With over 350 beds, a well-equipped and staffed laboratory, a modern X-ray department, five operating rooms, an operating amphitheatre, a teaching amphitheatre, a modern training school for nurses, with its dietetic and other laboratories, post-graduate courses in anæsthetics and in X-ray and operating room technique, a very complete out-patient department, with a well-trained social service staff, a special psychiatric clinic, a day nursery with classes in home economics for mothers, and a farm to supply its own products, it is doubtful if there exists a hospital with a more ambitious scheme or more modern development. It had reached the ultra-modern or social phase of hospital work long before most similar institutions had realised this new mission of the hospital. Its Report, which is for the year ending June 30, 1923, is admirable in nearly every respect, but we could wish that it had an index or table of contents.

